# Effects of cigarette smoke on endothelial function of pulmonary arteries in the guinea pig

**DOI:** 10.1186/1465-9921-10-76

**Published:** 2009-08-14

**Authors:** Elisabet Ferrer, Víctor Ivo Peinado, Marta Díez, Josep Lluís Carrasco, Melina Mara Musri, Anna Martínez, Robert Rodríguez-Roisin, Joan Albert Barberà

**Affiliations:** 1 Department of Pulmonary Medicine, Hospital Clínic-IDIBAPS, Barcelona, Spain; 2 Ciber de Enfermedades Respiratorias, Barcelona, Spain; 3 Biostatistic Unit, Department of Public Health, Universitat de Barcelona, Barcelona, Spain

## Abstract

**Background:**

Cigarette smoking may contribute to pulmonary hypertension in chronic obstructive pulmonary disease by altering the structure and function of pulmonary vessels at early disease stages. The objectives of this study were to evaluate the effects of long-term exposure to cigarette smoke on endothelial function and smooth muscle-cell proliferation in pulmonary arteries of guinea pigs.

**Methods:**

19 male Hartley guinea pigs were exposed to the smoke of 7 cigarettes/day, 5 days/week, for 3 and 6 months. 17 control guinea pigs were sham-exposed for the same periods. Endothelial function was evaluated in rings of pulmonary artery and aorta as the relaxation induced by ADP. The proliferation of smooth muscle cells and their phenotype in small pulmonary vessels were evaluated by immunohistochemical expression of α-actin and desmin. Vessel wall thickness, arteriolar muscularization and emphysema were assessed morphometrically. The expression of endothelial nitric oxide synthase (eNOS) was evaluated by Real Time-PCR.

**Results:**

Exposure to cigarette smoke reduced endothelium-dependent vasodilatation in pulmonary arteries (ANOVA p < 0.05) but not in the aorta. Endothelial dysfunction was apparent at 3 months of exposure and did not increase further after 6 months of exposure. Smoke-exposed animals showed proliferation of poorly differentiated smooth muscle cells in small vessels (p < 0.05) after 3 months of exposure. Prolonged exposure resulted in full muscularization of small pulmonary vessels (p < 0.05), wall thickening (p < 0.01) and increased contractility of the main pulmonary artery (p < 0.05), and enlargement of the alveolar spaces. Lung expression of eNOS was decreased in animals exposed to cigarette smoke.

**Conclusion:**

In the guinea pig, exposure to cigarette smoke induces selective endothelial dysfunction in pulmonary arteries, smooth muscle cell proliferation in small pulmonary vessels and reduced lung expression of eNOS. These changes appear after 3 months of exposure and precede the development of pulmonary emphysema.

## Introduction

Patients with chronic obstructive pulmonary disease (COPD) show intimal hyperplasia in pulmonary muscular arteries, which results from the proliferation of smooth muscle cells (SMCs), and an increased proportion of muscularized arterioles. In addition, pulmonary arteries of COPD patients show abnormal endothelium-dependent vascular reactivity [1,2]. Studies conducted in healthy smokers have also revealed intimal hyperplasia in pulmonary muscular arteries, which does not differ from that in patients with mild COPD [3]. Furthermore, endothelial function of pulmonary arteries in healthy smokers lies between that in non-smokers and COPD patients, thereby indicating that endothelial dysfunction is present at the origin of the disease [2]. The impairment of endothelial function results from changes in the expression and release of vasoactive mediators that also regulate cell growth [4]. Overall, these initial alterations may lead to persistent changes in the vascular structure and function that underlie the development of pulmonary hypertension in COPD [5].

Studies performed in animal models have attempted to reproduce some of the pulmonary alterations that occur in COPD [6,7]. Among these, the model of airflow obstruction resulting from exposure to cigarette smoke (CS) is probably the most satisfactory approach. Chronic exposure of the guinea pig to CS is a widely recognized model of COPD [6,8]. In this model, Wright et al. [9-12] have shown muscularization of small pulmonary vessels, which precedes the development of emphysema, as well as changes in the expression of vascular mediators. In a recent study performed in guinea pig precision-cut lung slices, short-term exposure to CS induced a delayed response to vasoactive agents in intracinar arteries [13]. Whether long-term exposure to CS in this animal model produces endothelial dysfunction in pulmonary arteries has not been addressed using the organ-bath methodology, which is the conventional method to assess endothelial function of pulmonary arteries in humans [2,14] and in animal models [15-18]. Furthermore, the extent to which changes in endothelial function are related to vessel remodeling and/or expression of endothelium-derived mediators remains to be established.

We hypothesized that in the guinea pig, long-term exposure to CS alters endothelial function, induces the proliferation of poorly differentiated SMCs in pulmonary vessels, and reduces the expression of endothelium-derived vasodilators, in a similar way as in humans [2-4]. Accordingly, the present study was addressed to investigate in the guinea pig the effects of chronic exposure to CS on the endothelial function of pulmonary arteries and the lung expression of endothelial nitric oxide synthase (eNOS), and to determine whether CS induces muscularization in small pulmonary vessels. We also studied the chronological sequence of the functional and morphological changes induced by CS on pulmonary vessels.

## Methods

### Animals and cigarette smoke exposure

Thirty-six male Hartley guinea pigs (Harlam Ibérica, Spain), each weighing 300 g, were given a diet of standard chow and water supplemented with vitamin C (1 g/L; Roche Pharma, Madrid, Spain) *ad libitum*. A group of 19 animals was exposed to the smoke of 7 research cigarettes (1R3F; Kentucky University Research; Lexington, KY, USA) per day, 5 days a week, using a nose-only system [6] (Protowerx Design Inc; Langley, British Columbia, Canada) for a period of 3 and 6 months (n = 6 and n = 13, respectively). Controls (n = 17) were sham-exposed during the same periods of time (n = 9 for 3 months, n = 8 for 6 months). Animals that died during the study were excluded from the sample size. All procedures involving animals and their care were approved by the Ethics Committee of the University of Barcelona and were conducted following institutional guidelines that comply with national (Generalitat de Catalunya decree 214/1997, DOGC 2450) and international (Guide for the Care and Use of Laboratory Animals, National Institutes of Health, 85-23, 1985) laws and policies.

### Endothelial function

After 3 or 6 months of CS exposure and 24 h after the last exposure, the animals were anesthetized with ketamine (50 mg/ml; 50 mg/kg. Pfizer Pharmaceuticals, Dun Laoghaire, Ireland) and xylazine (2%; 7 mg/kg. Bayer AG, Leverkusen, Germany) and the cardiopulmonary block was quickly removed to isolate a segment of the aorta and the main pulmonary artery. Arteries were cleaned of fat and connective tissue and cut into rings 3 mm in length. Two rings of the thoracic aorta and the left and right branches of the main pulmonary artery were placed in organ bath chambers (Panlab, Barcelona, Spain) filled with Krebs-Henseleit's buffer (containing (in mM) 118 NaCl, 24 NaHCO_3_, 11.1 glucose, 4.7 KCl, 1.2 KH_2_PO_4_, 1.2 MgSO_4_, 2.5 CaCl_2_), bubbled with a gas mixture of 21% O_2 _and 5% CO_2 _(pH 7.35–7.45) and kept at 37°C by an outer-water bath warmed by a recirculating heater. Ring preparations were attached to an isometric transducer (Panlab, Barcelona, Spain) and equilibrated for 1 h under a resting tension of 1.75 g for pulmonary artery and 2.3 g for aortic rings, as established in preliminary studies. After a period of stabilization, arteries were contracted with KCl (60 mM) to determine their viability and contractile capacity. On the basis of previous experience, arteries with contractions <1 g were considered not viable. All rings were pre-incubated with indomethacin (1 × 10^-5 ^M, Sigma Aldrich. St Louis, USA) 30 min before the experiments in order to inhibit the synthesis of cyclo-oxygenase products. Indomethacin was kept in the solution throughout the experiment. The rings were then contracted with norepinephrine (NE; 1 × 10^-7 ^to 0.2 × 10^-6 ^M, Sigma Aldrich.) to obtain a stable plateau of tension. Endothelial function was evaluated by adding adenosine-5'-diphosphate (ADP, Boehringer GmbH, Mannheim, Germany), an endothelial nitric oxide (NO)-dependent vasodilator, to the organ bath. One of the rings of the aorta and the left branch of the pulmonary artery were tested to cumulative concentrations of ADP (10^-9 ^to 10^-5^M). Response to cumulative concentrations of the exogenous NO donor, sodium nitroprusside (SNP; 10^-10 ^to 10^-5 ^M, Sigma Aldrich.), was also tested in the other two rings. To corroborate the endothelial function assay performed with ADP, all the procedures were repeated in the presence of *N*^G^-monomethyl-L-arginine (L-NAME; 10^-1 ^M, Sigma Aldrich.), an inhibitor of eNOS. Endothelium-dependent vasodilator responses were assessed by the maximal relaxation induced by ADP, the dose that caused 50% relaxation (EC_50_), and the area under the curve (AUC) [19] (Sigmaplot 10.0, Systat Software Inc, San José, CA, USA). Whereas EC_50 _is a single-point estimated value, the AUC is a summary measure obtained from all experimental points in the dose-response curve, providing a complete profile of vessel responsiveness. Each curve was evaluated by an observer without knowledge of the smoke exposure status.

### Histological Assessment

Explanted lungs were inflated with 4% formaldehyde at a constant pressure of 25 cm H_2_O during 24 h, and then embedded in paraffin. Histological examination was performed in 4-μm sagital sections stained with hematoxylin-eosin. The presence of emphysema was evaluated by measuring the mean linear intercept of alveolar septa in 20 randomly selected fields per slice using an image analysis system (Leica Qwin, Leica Microsystems Image Solutions Ltd, Cambridge, UK). Pulmonary vessels were analyzed in lung tissue sections stained with orcein. To assess the number of muscularized arterioles, all vessels with an external diameter <50 μm and with double elastic laminas were counted.

After the organ bath studies, all artery rings were fixed in 4% formaldehyde and cryo-embedded in optimal cutting temperature (O.C.T). Morphometric studies were performed in 4-μm slices of aorta and main pulmonary artery sections stained with elastin-Van Gienson. The external and internal elastic laminas were outlined and both total and lumen areas were computed using an image analysis system [2] (Leica Qwin). The area of the arterial wall was estimated as the difference between the total and luminal areas. Wall thickness was calculated by dividing the arterial wall area by the external perimeter of the artery [20].

### Immunohistochemical studies

The expression of desmin and α-actin in pulmonary vessels (< 50 μm) was assessed by immunohistochemistry using anti-α-actin and anti-desmin antibodies (Dako, Glostrup, Denmark). An avidin-biotin reaction was performed to amplify the signal. The immunoreactions to α-actin and desmin were quantified as the number of positive vessels per mm^2^. The intensity and extension of immunoreaction to desmin were also semi-quantitatively evaluated in a scale from 1 to 3 (for intensity, 1: low, 2: medium, 3: high; and for extension, 1: 0–25% of the vessel wall, 2: 26–75%, 3: > 75%).

### Real Time-PCR

Total RNA was extracted from lung tissue using TRIzol (Invitrogen, Paisley, Scotland, UK). For reverse transcription, 2.0 mg of total RNA was retrotranscribed using a high-capacity cDNA Archive kit (Applied Biosystems). Quantification of eNOS was done with real-time PCR using SYBR Green I chemistry (SensiMix (dT) DNA Kit, Quantance Ltd, Ballards Lane, London). Normalization of gene expression levels was performed by using β-actin as endogenous housekeeping gene. To generate a standard curve, 7-fold serial dilutions of each purified PCR product were used for templates. Primers were designed based on guinea pig (eNOS) sequence from GeneBank using specific software (Primer Express, Applied Biosystems, Foster City, CA.). Amplification was performed on Chromo4 thermocycler (MJ Research, BioRad, Hercules, CA), and each sample was run in duplicate. The identities of the amplified products were examined using 12% poly-acrylamide gel electrophoresis and melt curve analysis. The primer sequences for eNOS were 3'-AGCCAACGCGGTGAAGATC-5' and 5'-TTAGCCATCACCGTGCCC-3' and for β-actin 3'-ATATCGCTGCGCTCGTTGTC-5' and 5'-AACGATGCCGTGCTCAATG-3'.

### Statistics

To evaluate the effect of CS exposure on endothelial function, a general linear model [21] was fitted using time, group and time-by-group interaction as independent factors. The estimates of the factors were adjusted by the effect of contraction to NE by including it in the model. The significance of the independent factors was assessed by the common ANOVA F-test using the type-3 sum of squares. The adequacy of the model was checked by examination of the Pearson residuals. All other variables are expressed as mean ± standard deviation (SD) or as median and inter-quartile range depending on whether or not the variables followed a normal distribution. Comparisons between groups were performed by the Student t-test or Mann Whitney test. A p-value lower than 0.05 was considered significant.

## Results

Five of the 24 guinea pigs exposed to CS died during the study while no deaths occurred in the control group. The animals that died during the experiment were excluded from the analysis.

At the end of the study, animals showed normal behavior and activity. CS-exposed guinea pigs were smaller and had lower body weights than the controls (data not shown). The anesthesia was deep in all cases and anesthetic dosages were identical between controls and CS-exposed animals. The anatomical examination revealed no signs of respiratory infection or other major abnormalities in lung tissue.

### Vascular contractility

In pulmonary arteries, maximal contraction to KCl was greater in animals exposed to CS at 3 and 6 months than in controls (Table 1). Maximal contraction to NE was similar in all groups at all times of exposure.

**Table 1:** Vascular response of pulmonary artery

	3 months		6 months		ANOVA		
	
	Control (n = 9)	CS-Exposed (n = 6)	Control (n = 8)	CS-Exposed (n = 12)	Time	CS Exposure	Interaction
**Contraction**							
**KCl (60 mM), mg**	1825 ± 164	2318 ± 201	1673 ± 174	2694 ± 142	0.554	<0.001	0.134
**NE (10^-6^M), mg**	1043 ± 118	1058 ± 145	990 ± 125	895 ± 107	0.396	0.703	0.660

**Relaxation to ADP**							
**EC_50_, (-log [M] ADP)**	7.48 ± 0.07	7.28 ± 0.09	7.21 ± 0.07	7.15 ± 0.06	0.009	0.110	0.318
**AUC**^†^	7.71 ± 0.03	7.78 ± 0.04	7.83 ± 0.03	7.91 ± 0.03	<0.001	0.037	0.881

**Relaxation to ADP+L-NAME**							
**EC_50_, (-log [M] ADP)**	ND	ND	ND	ND	ND	ND	ND
**AUC**^†^	8.34 ± 0.05	8.30 ± 0.05	8.28 ± 0.05	8.27 ± 0.04	0.358	0.619	0.711

**Relaxation to SNP**							
**EC_50_, (-log [M] SNP)**	8.66 ± 0.12	8.66 ± 0.14	8.57 ± 0.14	8.36 ± 0.11	0.137	0.370	0.414
**AUC^†^**	7.52 ± 0.06	7.42 ± 0.07	7.59 ± 0.07	7.65 ± 0.05	0.022	0.816	0.201

Definition of abbreviation: NE: Norepinephrine; ADP: Adenosine diphosphate; SNP: Sodium nitroprusside; EC_50_: dose that causes 50% of relaxation; AUC: area under the curve; L-NAME: *N*^G^-monomethyl-L-arginine

Values are mean ± SEM

^†^log-transformed

ND: not determined

In the aorta, there were no differences in the maximal contraction to KCl between CS-exposed animals and controls at any time of exposure. The ANOVA revealed a time effect in the contractile response to NE (Table 2).

**Table 2:** Vascular response of aorta

	3 months		6 months		ANOVA		
	
	Control (n = 9)	CS-Exposed (n = 6)	Control (n = 8)	CS-Exposed (n = 12)	Time	CS Exposure	Interaction
**Contraction**							
**KCl (60 mM), mg**	3346 ± 290	3224 ± 336	3596 ± 290	3837 ± 237	0.144	0.755	0.537
**NE (10^-6^M), mg**	1347 ± 192	1621 ± 222	922 ± 192	1037 ± 157	0.012	0.341	0.682

**Relaxation to ADP**							
**EC_50_, (-log [M] ADP)**	7.10 ± 0.10	7.29 ± 0.12	6.97 ± 0.10	6.93 ± 0.08	0.038	0.588	0.260
**AUC**^†^	8.21 ± 0.04	8.15 ± 0.05	8.25 ± 0.04	8.23 ± 0.03	0.208	0.440	0.651

**Relaxation to ADP+L-NAME**							
**EC_50_, (-log [M] ADP)**	ND	ND	ND	ND	ND	ND	ND
**AUC**^†^	8.80 ± 0.06	8.85 ± 0.08	8.76 ± 0.07	8.67 ± 0.05	0.116	0.661	0.268

**Relaxation to SNP**							
**EC_50_, (-log [M] SNP)**	7.53 ± 0.15	7.26 ± 0.26	8.04 ± 0.13	8.15 ± 0.10	<0.001	0.837	0.286
**AUC**^†^	7.91 ± 0.07	7.99 ± 0.08	7.93 ± 0.07	7.88 ± 0.05	0.544	0.991	0.343

Definition of abbreviation: NE: Norepinephrine; ADP: Adenosine diphosphate; SNP: Sodium nitroprusside; EC_50_: dose that causes 50% of relaxation; AUC: area under the curve; L-NAME: *N*^G^-monomethyl-L-arginine

Values are mean ± SEM

^†^log-transformed

ND: not determined

### Endothelial function

**Figure 1:**
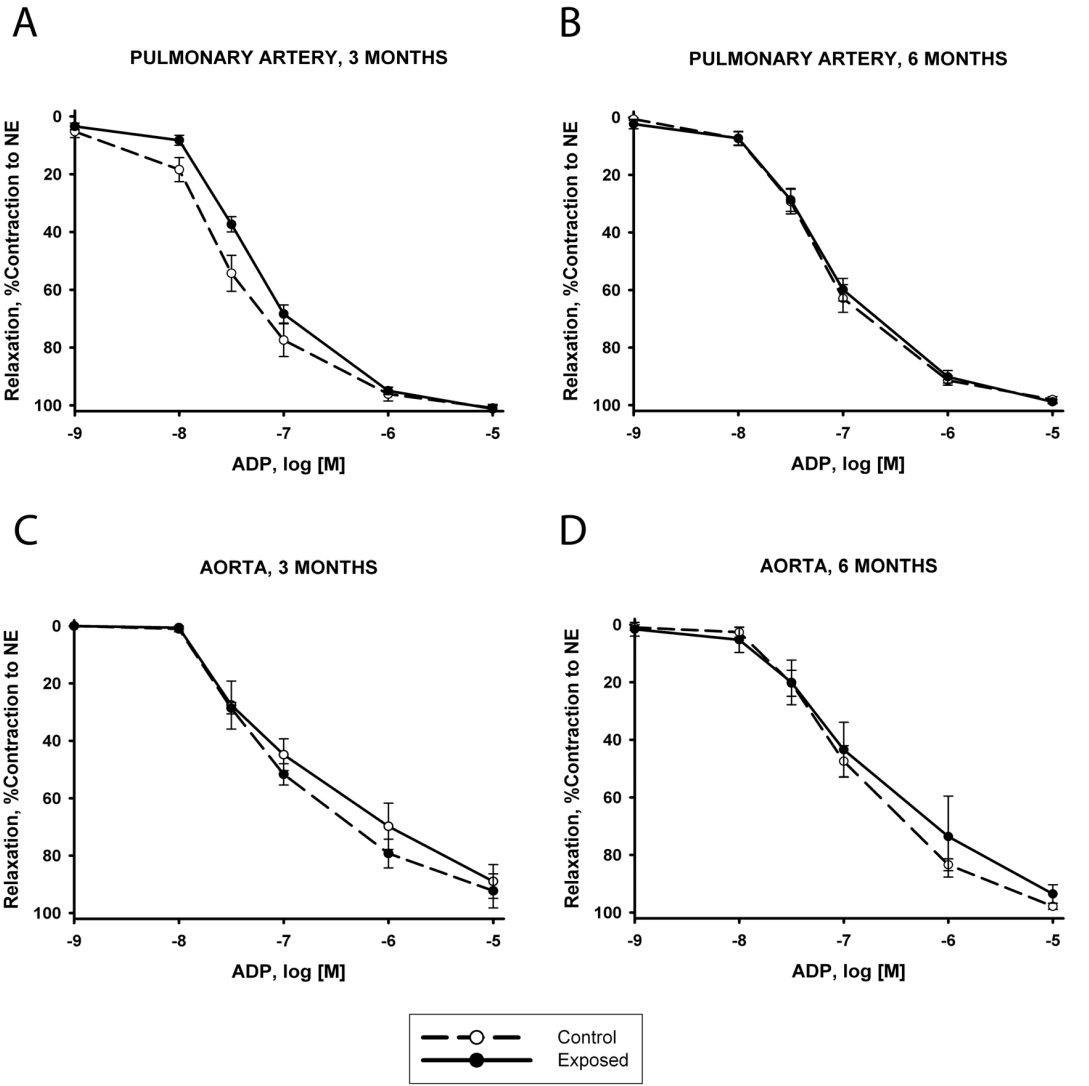
**Endothelial function of pulmonary and aorta arteries**. Relaxation of main pulmonary artery and aorta to cumulative doses of adenosine-5'-diphosphate (ADP) expressed as % of contraction to norepinephrine (NE). Upper panels show dose-response curves of pulmonary arteries in smoke exposed (continuous line) and control (dashed line) animals at 3 (A) and 6 months (B) of exposure. Lower panels show dose-response curves of aorta at 3 (C) and 6 months (D) of exposure. Values are mean ± SEM.

Guinea pigs exposed to CS showed a shift to the right in the dose-response curve of pulmonary arteries to ADP, as shown by a greater AUC and higher (less diluted) EC_50_, compared with control guinea pigs (Table 1, Figure 1). The ANOVA failed to show any interaction between CS exposure and time (Table 1), thereby indicating that reduced reactivity of pulmonary arteries in CS-exposed animals was independent of the length of exposure.

The ANOVA also revealed a marked effect of time on endothelium-dependent relaxation. Irrespective of whether the animals were exposed to CS, the endothelium-dependent vasodilatation of pulmonary arteries was lower at 6 months than at 3 months.

The maximal relaxation induced by ADP was close to 100% in all groups. When pulmonary artery rings were exposed to the competitive eNOS inhibitor, L-NAME, the relaxation induced by ADP was almost completely abolished (Table 1), indicating that ADP operated through the L-arginine-NO pathway. In all cases, pulmonary arteries reached maximal relaxation when they were assessed with SNP, an exogenous NO donor.

In rings of the aorta, no effect of CS exposure was found in any of the relaxation responses to the pharmacological agents that were tested (Table 2). Yet, the ANOVA revealed a time effect in both the endothelium-dependent and – independent relaxation responses.

### Morphological evaluation

**Figure 2:**
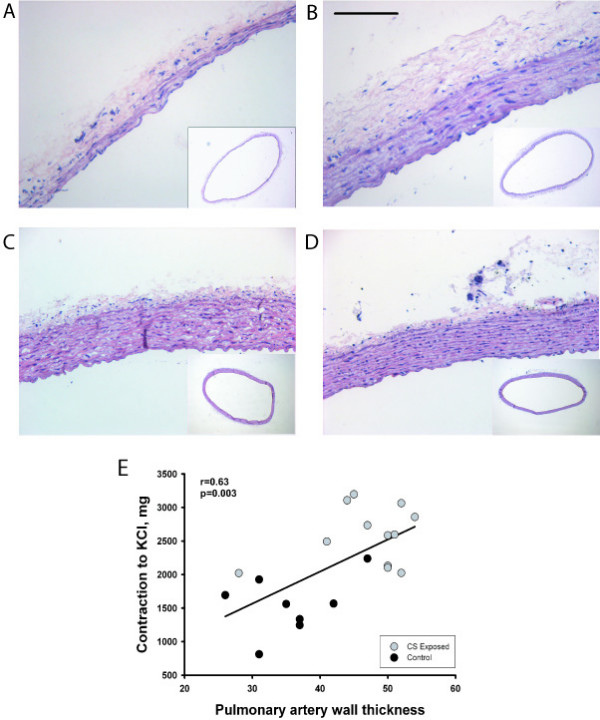
**Morphometry of pulmonary artery**. Hematoxylin-eosin stained sections of main pulmonary artery (upper panels) and aorta (lower panels) from control (A and C) and cigarette smoke (CS)-exposed (B and D) guinea pigs. Whereas, pulmonary artery of the exposed animal shows prominent thickening of the arterial wall, no difference in wall thickness is noticed in the aorta. Scale bar, 100 μm. (E) Correlation between the contraction to KCl and the wall thickness of the main pulmonary artery in control (black symbols) and CS-exposed (grey symbols) animals after 6 months of exposure.

The thickness of the walls of the right and left main pulmonary arteries was enlarged in CS-exposed animals (Figure 2A and 2B). Wall thickening of pulmonary arteries was due to both smooth muscle cell proliferation and elastic fiber deposition (data not shown). In contrast, the thickness of the aorta was not affected at either 3 or 6 months (Figure 2C and 2D, Table 3).

**Table 3:** Morphometric measurements in main pulmonary artery and aorta

		3 months		6 months	
		
		Control (n = 8)	CS-Exposed (n = 5)	Control (n = 8)	CS-Exposed (n = 13)
**Pulmonary artery**	**Diameter (mm)**	2.26 (2.10–2.49)	2.37 (2.11–2.45)	2.26 (2.13–2.39)	2.19 (2.15–2.40)
	**Wall area (mm^2^)**	0.26 (0.24–0.33)	0.38 (0.30–0.42)	0.27 (0.24–0.31)	0.35* (0.32–0.41)
	**Wall thickness (μm)**	38 (36–43)	50 (43–57)	35 (33–40)	51^‡ ^(45–53)

**Aorta**	**Diameter (mm)**	2.21 (2.14–2.30)	2.34 (2.17–3.80)	2.22 (2.07–2.32)	2.28 (2.24–2.36)
	**Wall area (mm^2^)**	0.58 (0.51–0.65)	0.77 (0.59–0.92)	0.50 (0.46–0.65)	0.63 (0.57–0.74)
	**Wall thickness (μm)**	82 (74–92)	96 (86–117)	77 (70–87)	88 (80–97)

Wall thickness was calculated as total area/external perimeter

Values are median and interquartile range

* p < 0.05

^‡ ^p ≤ 0.01

**Figure 3:**
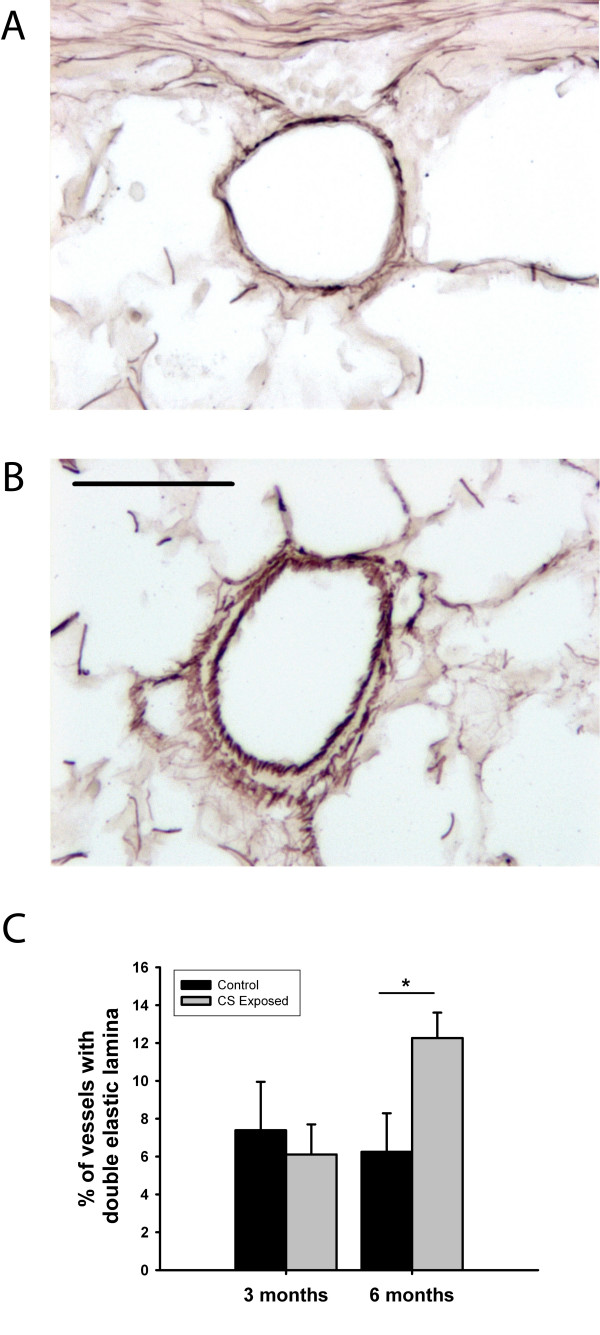
**Double elastic lamina presence in small intrapulmonary arteries**. Orcein stain of small pulmonary vessels in a control guinea pig (A) and an animal exposed to cigarette smoke (CS) (B). In the exposed animal a double elastic lamina is present, indicating full muscularization of the vessel. Scale bar, 50 μm. (C) Bar graph shows the number of vessels with double elastic laminas expressed as a percentage of the total number of vessels. The number of vessels with double elastic laminas was higher in guinea pigs exposed to CS for 6 months. * p < 0.05 compared with control group. Values are mean ± SEM.

The percentage of intra-pulmonary vessels with double elastic laminas increased 2-fold after 6 months of CS exposure compared to controls (12.3 ± 4.8 vs. 6.3 ± 5.7 respectively). This effect was not observed at 3 months (6.1 ± 3.9 vs. 7.4 ± 8.1 respectively) (Figure 3). There were no differences in the percentage of muscularized arterioles at 3 months of exposure.

### Immunohistochemical evaluation

**Figure 4:**
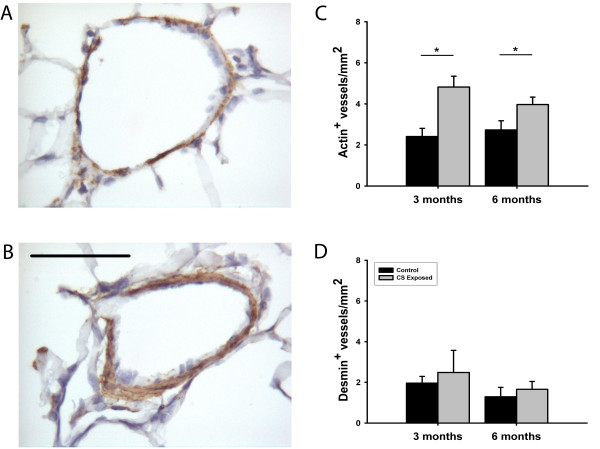
**Smooth muscle cell proliferation in small intrapulmonary arteries**. Immunohistochemistry for α-actin in small vessels of a control guinea pig (A) and an animal exposed to cigarette smoke (CS) (B). The vessel of the exposed animal shows a thicker wall with a strong immunoreactivity to α-actin. Scale bar, 50 μm. Bar graphs show the number of vessels/mm^2 ^with positive immunoreactivity to α-actin (C) and desmin (D) in control and CS-exposed guinea pigs, for 3 and 6 months of exposure. * p < 0.05 compared with control group. Values are mean ± SEM.

Immunohistochemical evaluation was performed in vessels with a diameter <50 μm. The number of vessels that were positive to smooth muscle α-actin was significantly greater in CS-exposed animals, at both 3 and 6 months of exposure (Figures 4A, B and 4C). In contrast, no changes were observed in the number of desmin-positive vessels at 3 or 6 months (Figure 4D), nor in the intensity or extension of protein expression (data not shown).

### Alveolar space size

An increase in the mean distance between alveolar septa was observed in animals exposed to CS for 6 months (control vs. exposed: 52 ± 8 vs. 59 ± 7 μm, p < 0.05). This finding is consistent with the presence of pulmonary emphysema. No differences between control animals and those exposed to CS for 3 months were found.

### Gene expression of eNOS

**Figure 5:**
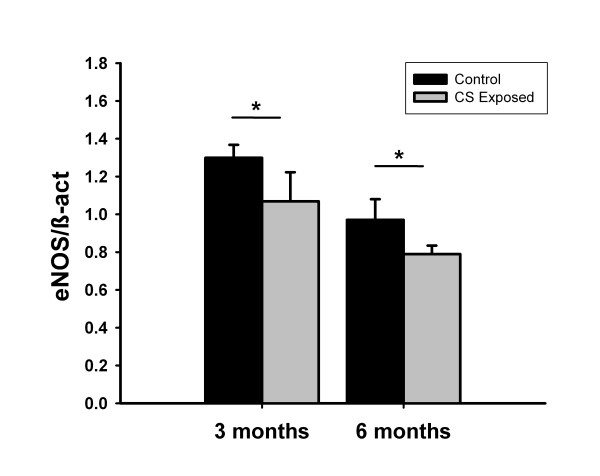
**Gene expression of eNOS in whole lungs evaluated by real time-PCR**. Bars show the expression of the eNOS gene, normalized by the expression of β-actin (β-act) gene. Compared with control guinea pigs, the expression of eNOS was reduced in animals exposed to cigarette smoke, both at 3 and 6 months of exposure. * p < 0.05, compared with control group. Values are mean ± SEM.

Gene expression of eNOS was evaluated by Real-Time PCR in whole lung homogenates and normalized by the expression of β-actin. Compared with control animals, eNOS expression was decreased at 3 and 6 months of exposure to CS (Figure 5).

### Correlation

The wall thickness of pulmonary arteries correlated significantly with the contraction to KCl (r = 0.63, p = 0.003) (Figure 2E). This correlation was not observed in aortas. There was no correlation between the endothelial function of the main pulmonary artery and the number of α-actin-positive intrapulmonary vessels or muscularized arterioles.

## Discussion

Our results show that guinea pigs chronically exposed to CS developed endothelial dysfunction in the pulmonary artery, which was already apparent after 3 months of exposure. In this period of time, animals exposed to CS showed reduced expression of eNOS in lung tissue and developed SMC proliferation in small intrapulmonary arteries. Longer exposure resulted in complete muscularization of small pulmonary vessels, as well as emphysematous changes in the alveolar spaces.

Endothelial dysfunction in pulmonary arteries was shown by a diminished response to the endothelium-dependent vasodilator ADP, which was abolished by the eNOS inhibitor L-NAME. Contrasting with this observation, no differences between groups were found in the endothelium-dependent relaxation of the aorta, thereby suggesting that CS exposure exerted a direct effect on pulmonary circulation. These results are in agreement with previous studies showing diminished endothelium-dependent relaxation in pulmonary arteries of smokers [2,4] and in guinea pigs after a short period of CS exposure [13]. Moreover, in the present study endothelial dysfunction preceded the complete muscularization of small intrapulmonary vessels (vessels with double elastic lamina), thereby supporting the hypothesis that in COPD endothelial dysfunction is an early event that antecedes pulmonary vascular remodeling [22]. The mechanisms by which CS impairs pulmonary endothelium remain to be established. Our results show a decrease in eNOS gene expression in the lungs of animals exposed to CS. This finding is in agreement with those of Su and co-workers [23], who demonstrated that eNOS is down-regulated in endothelial cell cultures exposed to cumulative doses of CS extract. The expression of eNOS is also reduced in pulmonary arteries of smokers and in patients with different degrees of COPD severity [4,24]. Accordingly, it is conceivable that CS might alter endothelium-dependent relaxation by down-regulating eNOS expression in pulmonary arteries of guinea pigs.

We also observed a marked effect of time on the endothelial function of pulmonary arteries. The vascular relaxation induced by ADP was lower at 6 months than at 3 months, irrespective of whether the animals were exposed to CS or not. The effect of time on vascular reactivity was also apparent in the aorta. Since the guinea pigs used in our study were in their growing period (mean weight increased by 231% at 3 months, and by 404% at 6 months), we consider that growth (or maturation) might affect vascular reactivity, in particular the endothelial function of pulmonary arteries, by mechanisms that have not been fully elucidated. Indeed, it has been observed that hormonal changes associated with sexual maturity may affect posttranscriptional and/or translational regulation of eNOS protein and result in lower plasma NO levels in adult male pigs, thereby exerting an effect on vascular function [25]. On the other hand, maturation also induces increased production of reactive oxygen species (ROS) in vessels, which, in turn, may impair vessel function as a result of decreased NO bioavailability [26-28].

We characterized the phenotype of the SMC responsible for vascular remodeling in guinea pigs exposed to CS by evaluating the expression of the intermediate filaments smooth muscle α-actin and desmin in small pulmonary vessels. Animals exposed to CS for 3 months showed an increase in the number of α-actin-positive vessels, which persisted after 6 months of exposure. In contrast, there were no differences in the number of vessels positive for the contractile filament desmin, either at 3 or at 6 months. Accordingly, CS-exposed guinea pigs showed proliferation of α-actin^+^/desmin^- ^SMC, which represent a subpopulation of less differentiated SMCs with synthetic capacity [5]. These results are consistent with those obtained in COPD showing that vascular remodeling is produced by the intimal proliferation of poorly differentiated SMCs [3]. The structural alterations in the pulmonary circulation of CS-exposed guinea pigs might be a consequence of changes in the synthesis and release of vasoactive and cell proliferative mediators, since endothelin and VEGF expression are increased in the arterial wall of remodeled vessels in animals exposed to CS [12]. After 6 months of exposure, we found an increased percentage of vessels with double elastic laminas. In the same experimental model, Wright et al. [9] also reported that the greater number of vessels with double elastic laminas was accompanied by an increase in pulmonary artery pressure (PAP). This observation suggests that muscularization of small vessels induced by CS exposure is associated with pulmonary hypertension. In keeping with this, we observed a wall enlargement in main pulmonary arteries after 6 months of CS exposure, which correlated with the contractility to KCl.

Smooth muscle cell proliferation in small vessels was already present at 3 months of exposure and preceded the development of emphysema. These findings confirm previous observations made by Yamato et al. [29] and are in agreement with studies performed in healthy smokers, who showed pulmonary vascular remodeling and endothelial dysfunction [2]. Although hypoxemia, which is associated with emphysema, is one of the strongest agents producing vasoconstriction and vessel remodeling, our results corroborate that CS exposure may have a similar effect on pulmonary vessels [30]. Nevertheless, the presence of hypoxemia in patients with COPD might exert a synergistic effect on the pathogenesis of pulmonary hypertension. Further studies are required to elucidate the potential synergism between cigarette smoke and hypoxia in this experimental model.

It is interesting to note that endothelial dysfunction and vessel remodeling associated with CS exposure affected selectively pulmonary arteries while the aorta remained unaltered. We consider this could be due to the fact that pulmonary vessels are exposed to greater concentrations of CS products, whereas the effects on the aorta might be eventually caused by products diffusing to the blood. We do not disregard that longer exposure to CS might exert an effect on the aorta or systemic vessels. Although, it is conceivable that longer exposure would also result in greater structural and functional damage of pulmonary vessels. Yet, it is noteworthy that in this experimental model pulmonary vessels develop changes after a short period of CS exposure that antecede changes in lung structure or systemic vascular involvement.

In conclusion, the guinea pig chronically exposed to CS develops endothelial dysfunction selectively in pulmonary arteries, without presenting changes in systemic arteries. This endothelial dysfunction is accompanied by reduced lung expression of eNOS and proliferation of poorly differentiated SMCs in small pulmonary vessels. These changes are already apparent after a relatively short period of CS exposure and precede the full muscularization of small pulmonary vessels and the development of emphysema. Results in this experimental model confirm that CS has direct and deleterious effects on the structure and function of pulmonary vessels that might contribute to the development of pulmonary hypertension in COPD.

## Competing interests

The authors declare that they have no competing interests.

## Authors' contributions

EF carried out the experimental work, participated in the analysis of the data and in the preparation of the manuscript. VIP was involved in the conception of the study, participated in its design and coordination, the analysis of the data and in the elaboration of the manuscript. MD provided support in the experimental work and data collection. JLC performed the statistical analysis and contributed to the analysis of the data. MMM carried out the RT-PCR experiments. AM contributed in the implementation of the study and in the immunohistochemical studies. RRR provided funding support and contributed in the analysis of the data. JAB conceived the study, raised funding support, participated in the design and implementation of the study, and in the revision of the manuscript for important intellectual content. All authors read and approved the final manuscript.
